# Transforming public health using value lens and extended partner networks

**DOI:** 10.1002/lrh2.10234

**Published:** 2020-07-04

**Authors:** Mohan R. Tanniru

**Affiliations:** ^1^ College of Public Health University of Arizona Phoenix Arizona USA

**Keywords:** partner networks, public health, transformation, value creation, value fulfillment

## Abstract

**Introduction:**

Organizational transformations have focused on creating and fulfilling value for customers, leveraging advanced technologies. Transforming public health (PH) faces an interesting challenge. The value created (preventive practices) to fulfill policy makers’ desire to reduce healthcare costs is realized by several external partners with varying goals and is practiced by the public (value in use), which often places low priority on prevention.

**Methods:**

This paper uses value lens to argue that PH transformation strategy must align the goals of all stakeholders involved. This may include allowing partners and the public to contextualize the preventive practices to see the value in near term and as relevant. It also means extending the number of partners PH uses and helping them connect with the public to seek shared alignment in shared goals of value fulfillment and value‐in‐use.

**Results:**

Using lessons from Covid‐19 and PH experience with partners in four different sectors: business, healthcare, public and community, the paper illustrates how PH transformation strategy can be implemented going forward.

**Conclusions:**

We conclude the paper with five distinct directions for future research to create and sustain value using the framework of learning health systems.

## 1 INTRODUCTION

Environmental, biological, and social systems have learned to live and thrive in complex ecosystems by continually moving between stability and instability to test, evaluate, learn, and adapt to varying changes outside their ecosystem.^1^, ^2^ Businesses have started to adapt to their own complex ecosystem of evolving technologies and changing customer value expectations by testing innovative value propositions with customers and fulfilling these propositions through exploration, evaluation, and adaptation using a mix of internal and partner resources.^3^, ^4^ Within healthcare, hospitals have begun to transform their operations to address complexity within their ecosystem, with accountability for patient care extending beyond the hospital walls and patients seeking care transition services on demand.^5^, ^6^ As a part of this transformation, hospitals have begun to co‐create value with customers using innovative ways to deliver care using a number of technologies, such as mobile apps, wearables, portals and tele‐health^7^, ^8^, ^9^]. Hospital have also begun leveraging partners, such as urgent and ambulatory care centers, home care centers, and social and community care providers in fulfilling the value.^10^, ^11^

Co‐creation of value is intended to align the goals of those who create value and those who experience this value, that is, value created meets value‐in‐use. Similarly, when businesses use partners to support value fulfillment, they are seeking to align their goals with those of their partners, that is, share in the benefits of revenues generated. Similarly, when hospitals co‐create value with patients, they seek to align the patients' goal to self‐manage their health and the hospital's goal to provide quality care at reduced costs. Similarly, when hospitals use external partners, they both seek to align their goals of sharing reimbursement dollars and reducing readmission costs.^12^ In both business and healthcare, the alignment in goals with partners leads to gaining access to information on value‐in‐use, so that new value can be created.

Public health (PH) (refers to departments, organizations, agencies, etc.) has a distinct challenge in aligning goals with customers and partners. Creating value by developing practices to prevent the spread of an infection or a disease condition calls for allocating funds by policy makers and investment in behavioral change by the public today, with value‐in‐use realized later. Similarly, PH uses several media and community partners to fulfill the value, and they as well as PH see value‐in‐use feedback for their effort from yet a different set of partners (state and federal agencies) later. In other words, PH, its customers, and its partners are all investing in value creation or value fulfillment, with no immediate feedback on their efforts with information on value‐in‐use. This makes it difficult to seek commitment in aligning goals that today are mostly implicit. This is in fact counter to the use of frequent value cycles (value creation, fulfillment, and value‐in‐use) needed to create and sustain value,^13^ and learning health systems research that advocates continual learning loops of data to knowledge, knowledge to performance and performance to data to tailor personalized care.^14^

All these time delays and lack of explicit goal alignment disappear when there is a health emergency such as Covid‐19. PH is creating value with goal alignment with the public in preventing the spread of infections and with policy makers by gaining access to needed monetary or staffing resources. It can summon partnerships in supporting value fulfillment with many non‐traditional partners, including hospitals, businesses, schools, and community and social organizations, etc. It is also able to gain feedback from these partners and the public to assess the effectiveness of preventive measures (ie, value‐in‐use) so they can adjust the value created (new value created). In some cases, it is even allowing partners to tailor the public health practices to suit their context. For example, staying under quarantine, maintaining social distancing, wearing a mask, washing hands, etc. have been adapted by partners based on their situation (eg, allowing knowledge workers to work from home, developing partitions for those who need to work in close quarters, staggering hours to reduce the number of workers in a plant at any given time, etc.).

In summary, PH is able to extend its partner network and allow partners and the population segment with whom they work to customize practices within their context so that they can help fulfill the value and provide value‐in‐use information for it to create new practices or value quickly, because there is an alignment in the goals of all stakeholders: policy makers, the public, and partners. While such alignment is developing in response to a health emergency, how can PH transform its operations going forward so it can sustain some of this goal alignment and partnership in normal times? Hence, the research question: *Can public health build a broader network of partners whose goals are aligned with it as they serve select population segments? Secondly*, *can partner engagement in customizing and fulfilling the value created lead to improved surveillance needed to analyze values gaps and create new value cycles?*

The paper is organized as follows. The next section looks at prior research on pandemics and the current Covid‐19 experience to understand the *extended partner network* that public health can leverage to support its transformation. The third section provides a strategy to aligns the goals of partners involved to tailor and fulfill the value created using examples of select nodes in different industry sectors. The fourth section concludes with a discussion of the capabilities PH needs to bring about such a transformation, along with directions for future research.

## 2 PRIOR RESEARCH AND DEVELOPMENT OF THE PUBLIC HEALTH NETWORK

Multiple research streams on pandemics over the last two decades have highlighted both the sources of information used by epidemiologists to understand the intensity of the spread and a number of models used to identify factors that have contributed to both the severity and transmissibility of viral infections. These factors are then used to develop policy guidelines for select institutions to limit economic and social disruptions. The challenge has always been, even during and after pandemics, to determine how effective are the value fulfillment strategies and what methods were used to gain real time feedback to assess the effectiveness of the selected practices. The rest of the section will elaborate on each of these from prior work on pandemics.

**FIGURE 1:**
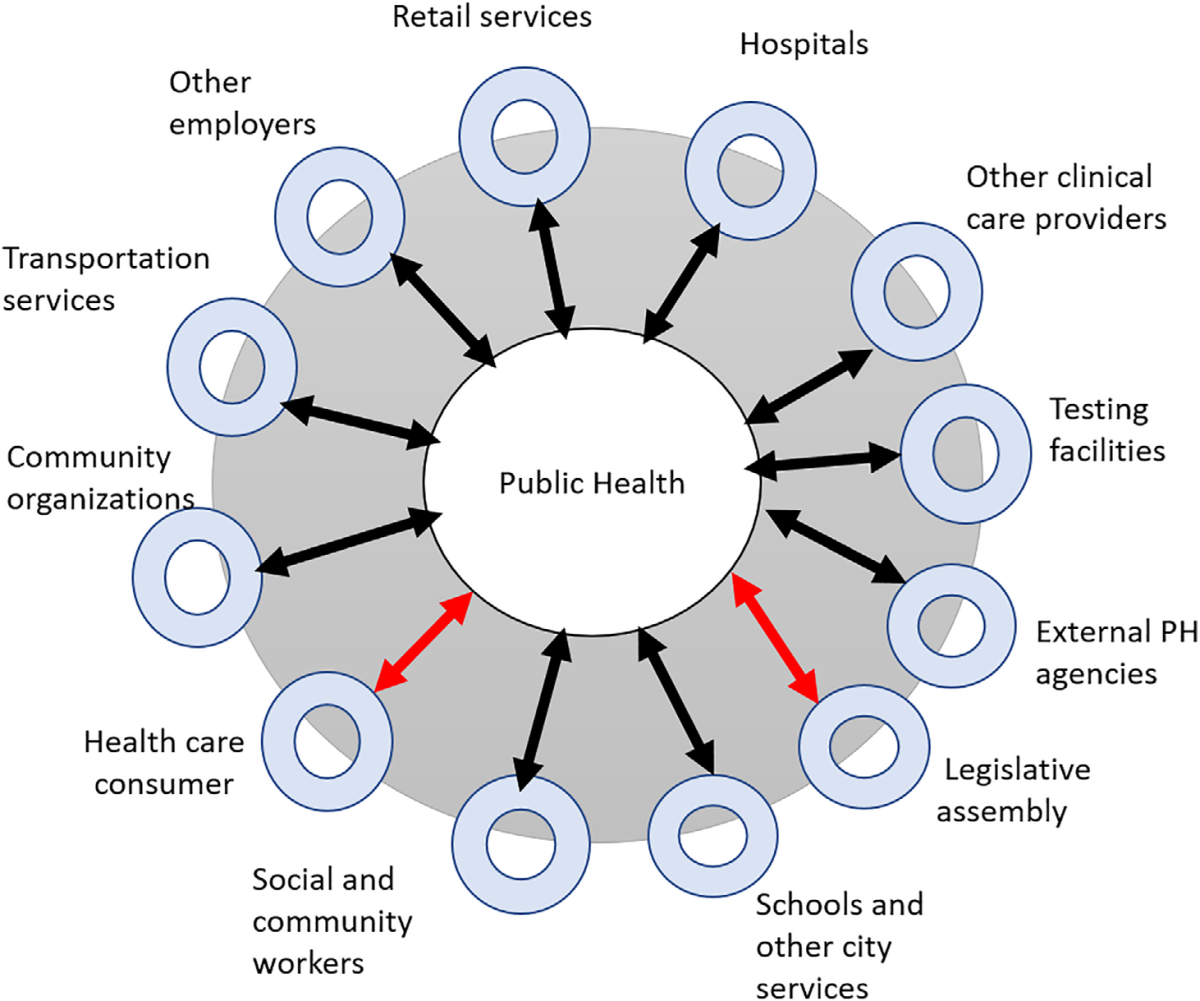
Public health extended network

Information from the mortality surveillance system was used to track deaths due to influenza outbreaks,^15^ and real time health insurance claims data including outpatient and emergency department visits can provide patient condition information post discharge.^16^ Inpatient data was used to capture information on patients' age and the presence of certain comorbidities and co‐diagnoses,^17^ as well as the breadth and depth of heterosubtypic immunity (ie, the immunity one gains with “seasonal” influenza) among certain population groups.^18^ Some of this clinical data is adjusted with other non‐clinical information, such as information on travel intensity,^19^ types of school closures that vary with grade levels and across regions after an infection outbreak occurs,^20^ quality of days or years (QALDs and QALYs) lost in work and school,^21^ and global travel characteristics of populations.^22^ In summary, surveillance methods used to gather intelligence phase of PH decision process includes both clinical and non‐clinical data. This data is generated from several nodes, as shown in Figure 1.

Diverse models are designed to analyze the data gathered during surveillance to identify factors that have contributed to the viral spread. The viral behavior within a human immune system and its various perturbations is complex, and non‐linear dynamic models are often used to determine factors contributing to the viral transmissibility.^23^ Other models were used to analyze data from the public: social media interactions to assess the pandemic spread^24^ and classification of Twitter messages to understand public trust on the practice message communicated.^25^ Models used within the clinical sector included those that analyzed the testing data to improve surveillance accuracy,^26^ resource utilization data in hospitals to assess the spread of an outbreak,^27^ the number of hospitalizations of children,^28^ and the impact of co‐morbidity on projected hospital length of stay, time to ventilation, and ventilation time.^29^ Other models in public and community sectors were used to assess the impact of community practices in schools and other regional institutions^30^ and the role of health inequities^31^ on the viral spread. The key for all these models is access to surveillance data in real time from several partner institutions, so that PH can assess value gaps or the effectiveness of its practice interventions.

Viral infections impact populations disproportionately (eg, pregnant women, children, or older populations during prior pandemics such as H1N1, SARS, Ebola, etc.) and can be transmitted differently (eg, exchange of bodily fluids as in HIV/AIDS or airborne and through human contact as in Covid‐19). Therefore, the policy and practice options selected should lead to specific guidance to different population segments based on their susceptibility to get sick such as pregnant women^32^ or those with respiratory infectious diseases.^33^ The guidance can be tailored to help institutions such as schools decide how to operationalize closures to reduce the intensity of viral spread.^34^ In addition, broader regional and national factors as well as community level factors must be considered before practice interventions are chosen for implementation.^35^ For example, New York City may have a different exposure to risk based on the density of its population and its movement using public transportation compared to regions like Montana where the population is spread out and use of personal cars for transportation is predominant. In summary, evidence‐based community level, assessment^36^ is needed to tailor practices before they are implemented.

From the above discussion, each node in Figure 1 is both an input for capturing the data needed for value creation and an output for practice implementation, with an opportunity to tailor the practice to its context. This is key for PH as it looks to learn from its pandemic experience to transform its operations. PH by itself cannot develop customized practices to each partner node. It can communicate the practice and let the partner node tailor this to align with its goals and that of the public it serves. For example, the business node can take the practice information and align it further to meet the business and employee goals by customizing the practice. Businesses may provide the option to employees to work from home, come on different dates, stay at a safe distance when they meet, etc. This is one of the reasons that the nodes in Figure 1 are shown as a double circle, representing both the partner and the population segment it serves. The public node is also shown separately, as some segments of the population are not directly connected with any single partner node. We will return to this in the next section.

Revisiting the research question, PH can rely on a several partners connected to various population segments to help fulfill the practices recommended, if it views the public as a collection of population groups that play various roles and some of these roles directly connect these population groups to specific partners (eg, employers when they work, patients when they go to hospitals, citizens when they see other public services, etc.). This leaves the second question: *How will the partner*'*s value creation and fulfillment help with the surveillance needed to analyze gaps and create new value cycles?* This is address in the next section.

## 3 SEEKING ALIGNMENT IN VALUE CREATION AND VALUE FULFILLMENT IN PUBLIC HEALTH

**FIGURE 2:**
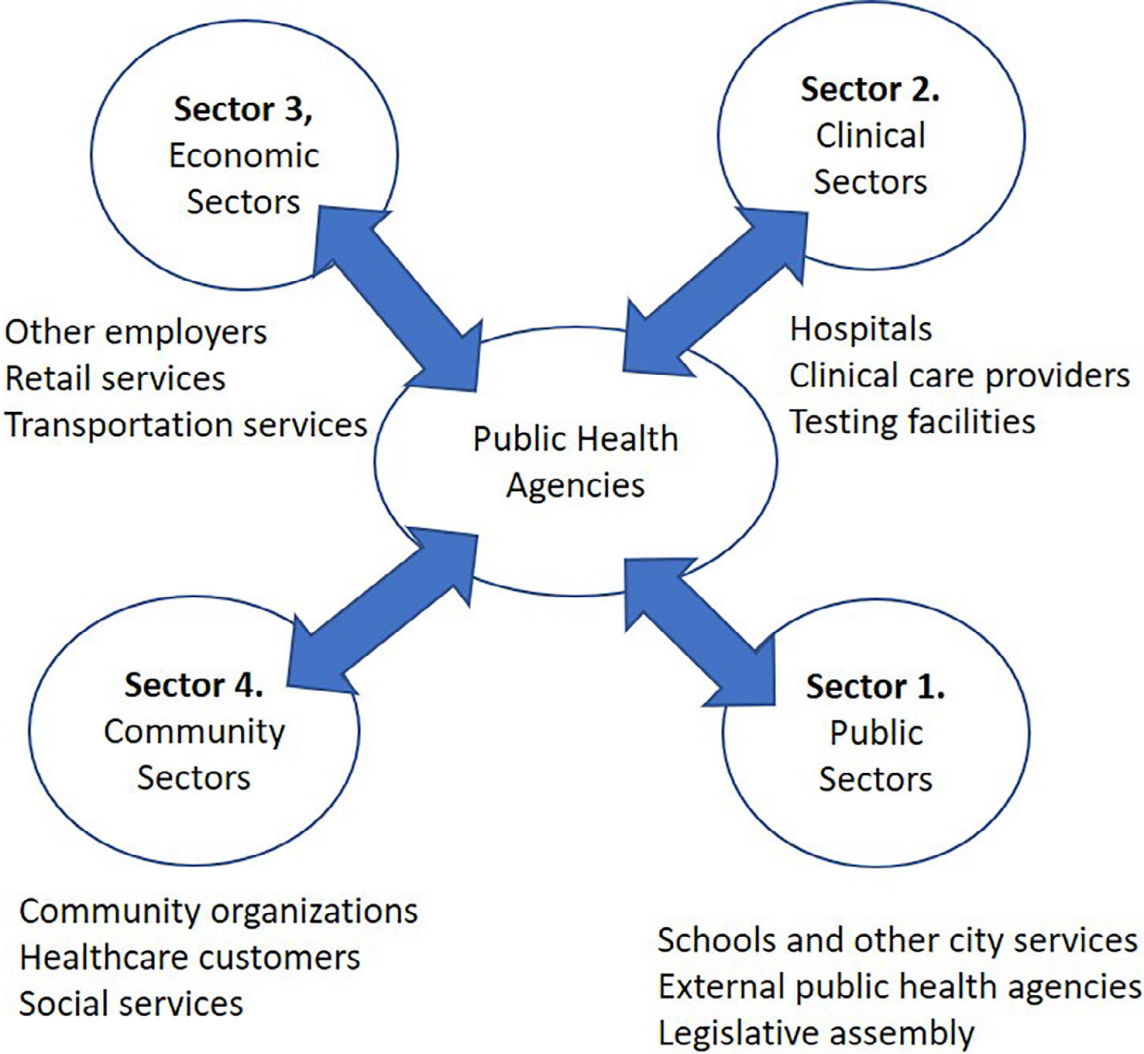
Clustering of nodes of the public health network

Service science research argues that in a service driven knowledge economy, organizations need to co‐create value propositions with their customers and fulfill these value propositions by leveraging internal resources and the resources of partners.^37^ Also, engaging consumers during value‐in‐use (ie, during consumers' use of the product purchased) can help discover gaps in value, leading to creating the next value cycle.^13^ In other words, organizations need to use successive value cycles to align their goals with the goals of their customers. Similarly, to fulfill the value crated, organizations and partners need to align their goals through shared resources. For PH to leverage its partners in value fulfillment and value‐in‐use, it needs to seek alignment with these partners. This is discussed in this section. For simplifying the discussion, Figure 2 segments the partners into four sectors. The economic sector includes institutions such as manufacturing and service industry partners, and the clinical sector includes health‐related institutional partners. The public and community sectors are represented in one sector, and the public sector includes other public sector services such as schools, public transformation, etc. as well as other PH agencies at various levels (state, country, and world). [Correction added on 17 July 2020 after first online publication: The preceding sentence has been revised from, “… and the political sector includes…”.] It also includes the other customers of PH.

### 3.1 Value creation and fulfillment with the clinical sector

Hospitals are interested in improving care outside a hospital to reduce readmission costs and use technology to help patients self‐manage their health condition. Many readmissions post‐discharge can be attributed to complications related to patient co‐morbidities such as diabetes and high blood pressure, and treatment plans often call for controlling these conditions through exercise, nutritional diet, stress reductions, etc. These are the same practices PH calls for to influence behavioral change. Such an alignment in goals can lead PH to share preventive practices to improve patient behavior as value created with hospitals. Hospitals, in turn, can share the information to patients, discharged or under their primary care, to fulfill the value created. Hospitals also can tailor these practices to patent groups and help assess any gaps in their adherence. This value gap in turn is communicated to PH for developing new preventive methods, possibly for specific patient groups. A public‐private partnership developed in Arizona [ASHLine]^38^ is an example of how a community partner was used directly by PH to create value and have physicians fulfill this value by referring their patients for smoking cessation. Yet, as discussed later in the community section, the value gap feedback for improvement in practices is not effectively sustained.

### 3.2 Value creation and fulfillment with the business sector

Keeping employees healthy by having them engage in preventive practices such as eating healthy food, walking when feasible while at work, and reducing family stress by having day care centers at work facilities are becoming a good business practice to improve employee morale, reduce absenteeism, improve productivity, and reduce healthcare costs. PH has many preventive practices to improve healthy behavior among population segments like the practices discussed above. However, there is limited effort to align the goals of employers and PH, even though there is significant effort in aligning the goals of hospitals, employers, and insurers for obvious reasons such as healthcare cost containment. In fact, the pandemic has highlighted the disadvantages of not sharing information when it came to employees working in meat processing plants, rural farms, and even urban cities where the public must use public transportation to get to their work.

While PH, hospitals, and businesses are not all going to be a part of the same enterprise creating and fulfilling value for employees, what is needed is an alignment in goals so PH can develop practices tailored to address the needs of seniors, women who are pregnant, children and youth, etc. and let businesses who serve these population groups fulfill the value created. For example, businesses that provide products or services to seniors, mothers who are pregnant, customers who use gyms, or schools that service young students can help fulfill the practice intervention and build customer loyalty. Similarly, businesses that employ similar population segments can tailor such practices to improve employee productivity, reduce absenteeism, and improve retention, even if it has no immediate impact on healthcare costs.

### 3.3 Value creation and fulfillment with community sector

Some of the population segments are not often affiliated directly with any single partner for several reasons. They may be geographically far remoted (eg, rural population, those who live in Indian reservations, etc.), too disenfranchised (eg, immigrant, indigenous, migrant, uninsured, etc.), or suffer health inequities along economic, social and cultural dimensions (eg, minorities in underserved areas, homeless populations, etc.). Both hospitals and PH often use social and community organizations to fulfill the preventive or care transition needs of such populations, and yet these organizations have limited resources and are aligned with the goals of their own funding agencies or donors. Even when their goals are aligned, they often lack the infrastructure to coordinate the value fulfillment (as their services tend to be voluntary) and assess information gathering for value in‐use. Yet, much of the disease burden during the pandemic and in normal times falls on this population segment, leading to significant healthcare costs. Four short use cases illustrate this challenge.

A non‐profit organization [HOPE],^39^ founded in 1998 as a community response to address homeless population needs, relies on its own partners, such as soup kitchens, temporary shelters, job training sites, etc., to help the homeless population transition to some semblance of recovery. While there is an implicit alignment in goals to create value for this population (prevention and recovering from the disease condition and support behavioral change) between hospitals, PH and HOPE, there is little infrastructure to share information or ability to tailor practices (as they have seen during current Covid‐19 with no easy away to fulfil social distancing and hand washing) and track value‐in‐use.

In the public‐private, partnership [ASHLine]^38^ discussed earlier, PH uses the partner to fulfill the prevention of smoking among public and has hospitals provide referrals to patients to visit ASHLine.^38^ With partners using a mix of technologies including fax, phone calls, and some database technology to gather and share information, it becomes a challenge to communicate and adapt practices. For example, during Covid‐19, leveraging this partner to share or tailor practices for patients, who most likely have several co‐morbid conditions (heart and respiratory conditions) that increase their susceptibility to infection or complications, would have been helpful.

A mobile unit [Mobile]^40^ prescreens Hispanic populations in rural and underserved areas in Arizona for diabetes and other health conditions and provides referrals to physicians for follow‐up and setting up appointments for free clinics, when feasible. Despite the partner's effort, there is no direct alignment of goals today between PH and the mobile unit on assessing value gaps and no alignment of goals between physicians and the mobile unit in value fulfillment (follow through on referrals). Again, a lack of infrastructure and coordination among PH and partners left many questions posed by population about Covid‐19 answered by staff using their limited resources.

Several partners provide support to caregivers of cancer patients of Hispanic origin (Abrazo), older patients who are considered highly susceptible for readmission (Partners in care), and patients with dementia, cancer, etc. (Hospice of the Valley). However, these are independent partners, funded by various agencies, with no infrastructure to support value creation and fulfillment. Alignment of these partners with the PH, hospitals, and donors is ad hoc at best. With the growing use of tele‐health to enable virtual caregiver support, there are opportunities to improve the sharing of information in support of both value creation and assessment.

These examples simply highlight the complexities PH faces as it tries to transform itself to create and sustain value using the resources of its many partners. All these cases implicitly connect these community care providers and the population groups they serve with the PH and the clinical sector. With the growing alignment of goals of both PH and the clinical sector to manage the population for preventive health and transition of care, this sector provides an important opportunity to reduce healthcare costs, if community partners are given the ability to tailor practices to address the needed of population within their ecosystem and the infrastructure to share information.

### 3.4 Value creation and fulfillment within the public sector

The public sector incudes other public services besides PH, PH agencies/departments in other regions and countries, as well as policy makers. We will look at the first two here.

*Public sector services:* When the Centers for Disease Control (CDC)^41^ developed specific guidelines after the 2009 H1N1 pandemic, it included many PH partners such as state and local public health officials, schools, patients who are supported by community organizations besides hospitals, daycare centers, and public transportation companies. Each of the partners within the government can help fulfill the value created by PH in normal times. For example, agencies that supervise schools that want to improve the quality of lunches served, transport agencies that seek to provide accessibility to seniors, community organizations that seek to address food and home insecurities, etc. can help fulfill value created by PH on eating healthy food to reduce obesity in children, reducing social isolation among seniors, improve infection control using flu shots and immunizations, etc. While some of these occur today, a lack of real time information sharing often contributes to the inability to tailor practices (eg, leveraging influential members of the society to impress upon measles vaccination).

*PH agencies:* While the long‐term ecological and epidemiological dynamics of viral infections globally may follow very simple rules even for highly mobile populations,^23^ the spread of viral infections is often dictated by contextual factors associated with the community in which policy makers operate. The key decisions PH policy makers must make during pandemics relate to how large a response to mount, which control/intervention measures to implement, and for whom and when.^42^ In addition to historical data gathered from prior pandemics or epidemics at various levels of granularity, real time data that comes from various community and regional nodes is critical for community level response. For example, this data may include confirmed cases, syndromic surveillance, outbreak investigations, serological data, and clinical cases, as well as data for customer surveys and hospitalizations from a regional level and viral surveillance data from around the globe. PH agencies need a shared repository for real time capture of this information to contextualize practices in support of their communities. Coivd‐19 has shown how multiple technologies are being used including Google searches^43^ and spatial analysis [^44^] to assess the rate at which virus is spreading, but the same can be used to address other health conditions (eg, mental health, drug addition, obesity) and how they are addressed in different countries with many resource constraints (reverse innovations^45^ for possible adaption.

This section highlighted the need for PH, as a part of its transformation, to use a broader lens to seek partners who can make a preventive practice that is beneficial in the long term become personal and relevant in the near term. By allowing partners to adapt and help fulfill the value created, they can help gather real time surveillance of value in use before new practices are developed to address changes in the diverse ecosystems of the public.

## 4 CONCLUSION AND DIRECTIONS FOR FUTURE RESEARCH

Using prior research and experience from recent pandemics, the paper articulates how PH transformation can begin by reaching out to several partners that touch distinct population groups. By seeking alignment of PH goals with partner goals, PH can let the partners support value fulfillment, potentially by tailoring the value created to their specific context. The data gathered in real time from these partners, combined with other aggregated public health data, can then be used for analysis of the value gaps before new value is created.

**FIGURE 3:**
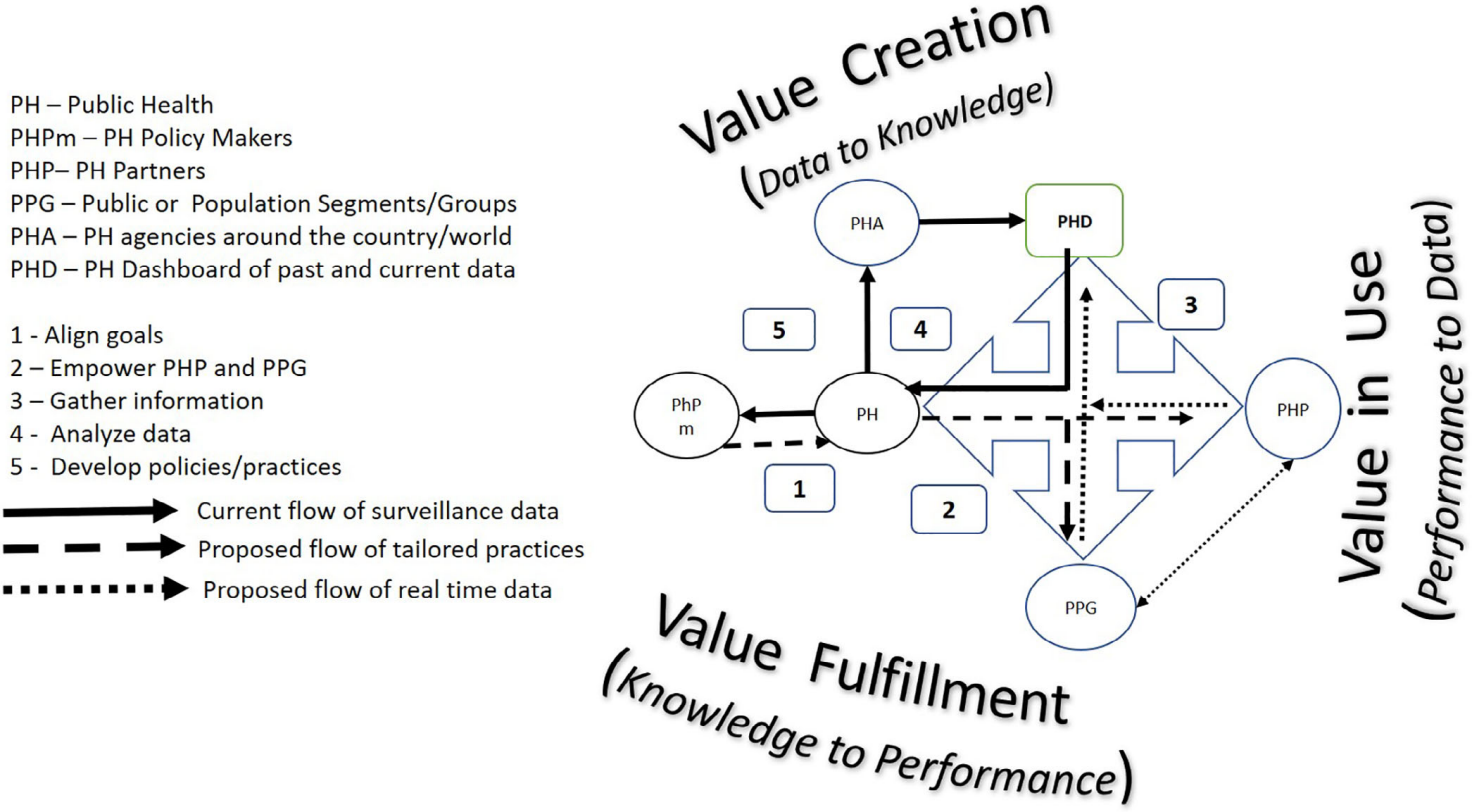
Directions for future research

Figure 3 below identifies five distinct areas for future research if PH is to continue to be service driven and learning focused.

- *Align goals* with partners, in emergencies or normal times, is about balancing health and economic risks among the stakeholders involved. These risks can be tangible (eg, loss of income, disruption to family life, health related complications, spread of infections) and intangible (eg, lost experience, disruption to work‐flow, depression, anxiety, isolation, etc.). Also, risks vary depending on partner‐population segment cohesion. If the segment is cohesive and distinct as in people working in meat packing or manufacturing firms, these partners may be given an opportunity to tailor practices to address these trade‐offs at their local context. On the other hand, airlines, and population segment that travels are not cohesive, and PH may set the guidelines based on the need for value fulfillment by all that travel. Use of multi‐criteria decision‐making and a mix of quantitative and qualitative decision models may be needed in selecting partners allowed to tailor practices.
- *Empower partners and public* to fulfill practices. Inverse care law^46^ may apply here, as practices may never reach the population segment the partner is trying to reach. This is often attributed to the partner not knowing how to reach the population group they are asked to support or have trust related challenges with the group. For example, perceptions that the data may be used for surveillance, performance evaluation or assessing penalties, shared with insurers or peers, etc. can lead to distrust. Research is needed on how to incentivize the partners and population groups using incentives or gamification to work together and develop privacy and security protocols to address information sharing.
- *Gather information* in as close to a real time as feasible from partners on population adherence to practices developed in value fulfillment. Besides addressing the privacy and security issues in sharing some of this information, PH and partners need to recognize that diverse technologies are used by these population groups to share the value‐in‐use information for analysis. An external third party may be used to reduce distrust and provide the information in anonymized and in aggregate form for partners and PH. Information architectures specific to such data collection^47^ or distributed architectures such as blockchain may be explored to support some of this communication and collaboration.
- *Analyze data* collected from each partner node and consolidate this with other regional and national data to assess health trends or infection spread. Since the timing, accuracy, and security/privacy of information shared can vary for clinical and non‐clinical data shared, new approaches are needed to combine this data. There is need for data cleansing and normalization prior to analysis. Advances in data science may be relevant in support of some of this analysis. Some of the data collected here may be contextualized for use in developing policies as well as storing them for future use.^45^
- *Develop policies and practices* to address the spread of infections or improve health conditions. The learning from previous value fulfillment effort should become a part of the shared knowledge for future use by all PH agencies. CDC^41^ guidelines post H1N1 is one example of sharing prior learning using a WHO^48^ pandemic framework,^49^ but how well these are available in real time for today's pandemic is unclear. Also, learning from prior explorations (eg, heuristics, use cases, expert opinions, etc.) developed possibly by autonomous population agents^50^ can help PH tailor prior policy or practice options to current context quickly. AI and machine leaning methods as well as intelligent agent technology may be useful here.

In summary, this paper suggests that PH take a broader value creation and fulfillment lens to support its transformation. The breadth seen through such a lens includes the use of multiple partners who align their goals and the population groups they support and the goals of PH to tailor health practices and support their implementation. The broader lens allows gathering of diverse data from these partners and the health conditions of the population groups to assess gaps between value created and value in use (customer adherence) to create new policies/practices. Similarly, the broader lens helps PH to view public as multiple population groups, each needing a more narrowly focused approach to improve their adherence to health behavior and reduce healthcare costs. As stated at the beginning, PH needs to transform citizen's perspective on prevention as personal for their immediate engagement.
